# Micronuclei and Nuclear Buds Induced by Cyclophosphamide in *Crocodylus moreletii* as Useful Biomarkers in Aquatic Environments

**DOI:** 10.3390/ani11113178

**Published:** 2021-11-07

**Authors:** Ana Lourdes Zamora-Perez, Jaime Luna-Aguirre, Guillermo Moisés Zúñiga-González, Olivia Torres-Bugarín, Blanca Miriam Torres-Mendoza, Martha Patricia Gallegos-Arreola, Ramón Guillermo Ortiz-García, Juan Ernesto Gutiérrez-Sevilla, Belinda Claudia Gómez-Meda

**Affiliations:** 1 Departamento de Clínicas Odontológicas Integrales, Instituto de Investigación en Odontología, Centro Universitario de Ciencias de la Salud, Universidad de Guadalajara, Guadalajara 44340, Jalisco, Mexico; lourdes.zamora@academicos.udg.mx; 2 División de Tecnología Ambiental, Universidad Tecnológica de Puebla, Puebla 72300, Mexico; jaime.luna@utpuebla.edu.mx; 3 Laboratorio de Mutagénesis, División de Medicina Molecular, Centro de Investigación Biomédica de Occidente, Instituto Mexicano del Seguro Social, Guadalajara 44340, Jalisco, Mexico; mutagenesis95@hotmail.com (G.M.Z.-G.); rguillermo.ortiz@alumnos.udg.mx (R.G.O.-G.); 4 Departamento de Investigación, Facultad de Medicina, Universidad Autónoma de Guadalajara, Guadalajara 44100, Jalisco, Mexico; olivia.torres@edu.uag.mx; 5 Laboratorio de Inmunodeficiencias y Retrovirus Humanos, Centro de Investigación Biomédica de Occidente, Instituto Mexicano del Seguro Social, Guadalajara 44340, Jalisco, Mexico; blanca.torresm@imss.gob.mx (B.M.T.-M.); juan.gsevilla@alumnos.udg.mx (J.E.G.-S.); 6 Departamento de Disciplinas Filosófico, Metodológicas e Instrumentales, Centro Universitario de Ciencias de la Salud, Universidad de Guadalajara, Guadalajara 44340, Jalisco, Mexico; 7 División de Genética, Laboratorio de Genética Molecular, Centro de Investigación Biomédica de Occidente, Instituto Mexicano del Seguro Social, Guadalajara 44340, Jalisco, Mexico; marthapatriciagallegos08@gmail.com; 8 Departamento de Biología Molecular y Genómica, Instituto de Genética Humana “Dr. Enrique Corona Rivera”, Centro Universitario de Ciencias de la Salud, Universidad de Guadalajara, Guadalajara 44340, Jalisco, Mexico; 9 Departamento de Microbiología y Patología, Centro Universitario de Ciencias de la Salud, Universidad de Guadalajara, Guadalajara 44340, Jalisco, Mexico

**Keywords:** erythrocytes, crocodiles, genotoxicity, micronuclei, nuclear buds

## Abstract

**Simple Summary:**

Crocodiles are territorial reptiles that are exposed to both aquatic and terrestrial environments. Analyzing their state of health and the environment in which they live is essential to detect changes that could affect them by exposure to agents that damage their genetic material, putting their health or other species that share said habitat, including humans, at risk. There are several ways to determine exposure to harmful agents, a very simple and direct one is to analyze the nuclei of blood cells under a microscope, a test that can also be carried out directly in their habitat and that gives rapid results on the effects of agents on those at that moment they are exposed. This study demonstrates the possibility of analyzing the blood of *Crocodylus moreletii* to quickly analyze its exposure to toxic agents in a sample of its blood by evaluating two abnormal structures in its cells under the microscope, demonstrating that evidence of damage can be observed only by analyzing a drop of their blood.

**Abstract:**

Micronuclei (MN) are used to assess genotoxic exposure, whereas nuclear buds (NBs) have been linked to genotoxic events. *Crocodylus moreletii* was studied to identify MN and NBs. Three groups were formed: Group 1 (water) and groups 2 and 3 (7 or 10 mg/kg of cyclophosphamide). A drop of blood was obtained daily from the claw tip at 0 to 120 h. Spontaneous micronucleated erythrocytes (MNEs) and erythrocytes with nuclear buds (NBEs) were counted. The frequencies of micronucleated young erythrocytes (MNYEs) and NB young erythrocytes (NBYEs) were evaluated, including the ratio of young erythrocytes (YE)/1000 total erythrocytes. No significant differences were observed in the YE proportion on sampling days; group 1 did not show differences for any parameter, whereas group 2 showed significant differences in MNEs and NBEs, and group 3 showed differences in NBEs and NBYEs. Some mitotic activity in circulation was observed in YEs. In conclusion, NBEs could be a more sensitive biomarker to genotoxic damage than MNEs. The identification of these biomarkers leads us to propose *Crocodylus moreletii* as a possible environment bioindicator because these parameters could be useful to analyze the in vivo health status of these reptiles and for biomonitoring genotoxic pollutants in their habitats.

## 1. Introduction

Aquatic environmental pollution is a serious and growing problem that affects all human activities. Aquatic ecosystems are regularly the ultimate recipient of many of the pollutants produced by natural and anthropogenic sources [1]. Chemical contaminants with genotoxic and carcinogenic potential in aquatic environments are a serious concern because they constitute a threat to aquatic and terrestrial life [2,3]. Nonetheless, reliable and practical methods that detect the presence of genotoxic contaminants are scarce [4]. The micronucleus assay is commonly used for evaluating structural and numerical chromosomal aberrations caused by clastogenic and aneugenic agents [5,6,7,8], and it was originally developed in mammals [5,9,10,11,12]; however, it has been successfully adapted to other non-mammalian organisms, such as fish erythrocytes [13], amphibian larvae [14,15], and the shed skin of salamanders [16], among others.

Nuclear protrusions termed “buds” have been described as potential biomarkers of genotoxicity [17,18,19,20]. In leukocytes [17,18] and erythrocytes of parrots [20], nuclear buds (NBs) are also markers of genotoxicity, and they can be observed in preparations used to evaluate the presence of micronuclei (MN). In culture of human and pig lymphocytes, the number of NBs increased with the administration of mitomycin-C, so it was proposed that NBs could be used as markers of exposure to genotoxic agents [17]. In this context, the presence of NBs could serve as a complementary measure to evaluate in vivo and in situ genotoxicity in species with nucleated erythrocytes.

To monitor genotoxic agents by counting the increase in MN and/or NBs within a population or among different populations as these nuclear abnormalities occur spontaneously, it should be noted that the species used must present MN and/or NBs in a sufficient quantity to be able to make comparisons with populations exposed to genotoxic pollutants in their environment.

In previous studies, a greater sensitivity has been observed in nuclear abnormalities compared to MN; that is, with less damage, an increase in nuclear abnormalities can be observed before the increase in MN [21,22]. There are other nuclear abnormalities that can be identified with these tests, but in this study, cyclophosphamide (CP) administered at low doses to crocodiles of the species *C. moreletii* has been used to identify and analyze only MN and NBs as markers of genetic damage.

CP has commonly been used as a positive control in genotoxicity studies because it is a known agent that damages genetic material. Cyclophosphamide belongs to a group of compounds called alkylating antineoplastic drugs, but it is inactive until it reaches the liver, where it is converted into active metabolites; it is a compound used in cancer therapies and has the characteristic of producing DNA strand breaks; that is, it is a clastogenic agent [23,24,25].

It is important to mention that other species of reptiles have been proposed to genotoxic monitoring using a micronucleus test and comet assay, such as turtles including, *Podocnemis expansa* and *Trachemys callirostris* [25,26], and also the broad-snouted caiman (*Caiman latirostris*) has been proposed for studies of this type through the micronucleus and nuclear abnormalities assays [27,28], which is an organism related to the species selected for this study (*Crocodylus moreletii*). The aim of this study was to assess the usefulness of MN and NB assays in peripheral blood nucleated erythrocytes from *C. moreletii* for in vivo biomonitoring of genotoxic pollutants and could be useful to analyze the in vivo health status of these reptiles in their habitats.

## 2. Materials and Methods

### 2.1. Animals

Fifteen swamp crocodiles (*C. moreletii)* from the “Centro para la Conservación e Investigación de la Vida Silvestre Guadalajara” (The Wildlife Research and Conservation Center-SEMARNAT) located in Guadalajara, Jalisco, Mexico, were used in this study (authorized by the National Secretary of Management for Environmental Protection SGPA/DGVS/04026, SGPA/DGVS/01373/06). The average age of *C. moreletii* was 8.0 ± 1.0 months, weight 70.3 ± 16.5 g, and length 32.2 ± 4.0 cm at the beginning of the experiment. Before experimental observations, alligators were acclimated to laboratory conditions for at least 9 days.

The crocodiles were handled in accordance with institutional guidelines and National and International Institutes of Health regulations for the humane treatment of research animals. Considering that crocodiles are poikilothermic organisms, they were housed in a container with a thermal insulation system to regulate and control the temperature at 30 °C. This is the average water temperature in the Alcuzahue Lagoon (Colima, México), the natural habitat of the crocodiles that were captured by veterinarians from The Wildlife Research and Conservation Center-SEMARNAT, with permission from the national environmental authorities, because they were previously introduced into this lagoon and were displacing the native species *C. acutus*. Crocodiles were fed with neonatal rats and mice euthanized by decapitation, which were provided by the laboratory animal facilities from the Centro de Investigación Biomédica de Occidente, Instituto Mexicano del Seguro Social.

### 2.2. Study Groups and Induction of Genotoxic Damage

A total of 3 study groups were formed with 5 crocodiles each: group 1 was the negative control, which only received water; group 2 received 7 mg/kg of CP (Sigma, St. Louis, MO, USA; CAS No. 6055-19-2); and group 3 received 10 mg/kg of CP for two consecutive days (14 and 20 mg/kg of total weight). In all cases, the oral dosage was prepared daily and administered through an orogastric cannula of 8 cm in length, adjusting to a final volume of 0.5 mL.

### 2.3. Blood Collection and Sample Preparation

A drop of peripheral blood from each crocodile was obtained from the tip of the claw with a nail clipper at 0, 24, 48, 72, 96 and 120 h, and it was smeared on two pre-cleaned and pre-coded microscope slides. The smears were air-dried and fixed in absolute ethanol for 10 min and stained with acridine orange (CAS No. 10127-02-3; Sigma, St. Louis, MO, USA) [23].

### 2.4. Sample Analysis

All slides were coded before microscopic analysis. Samples were evaluated by the same researcher, and they were scored manually using an OLYMPUS BX51 epifluorescence microscope (Olympus, Tokyo, Japan) and an oil-immersion objective (100×). The number of spontaneous micronucleated erythrocytes (MNEs) and the nuclear bud formation in erythrocytes (NBEs) were determined in a total of 10,000 erythrocytes (TEs). The frequencies of micronucleated young erythrocytes (MNYEs) and NB young erythrocytes (NBYEs) were also determined in 1000 young erythrocytes (YEs); the proportion of YEs in 1000 TEs was counted to determine a decrease in the cellular division as a cytotoxic effect. Acridine orange stained the YEs red or orange, while normochromatic (mature) erythrocytes were dark green.

**Figure 1:**
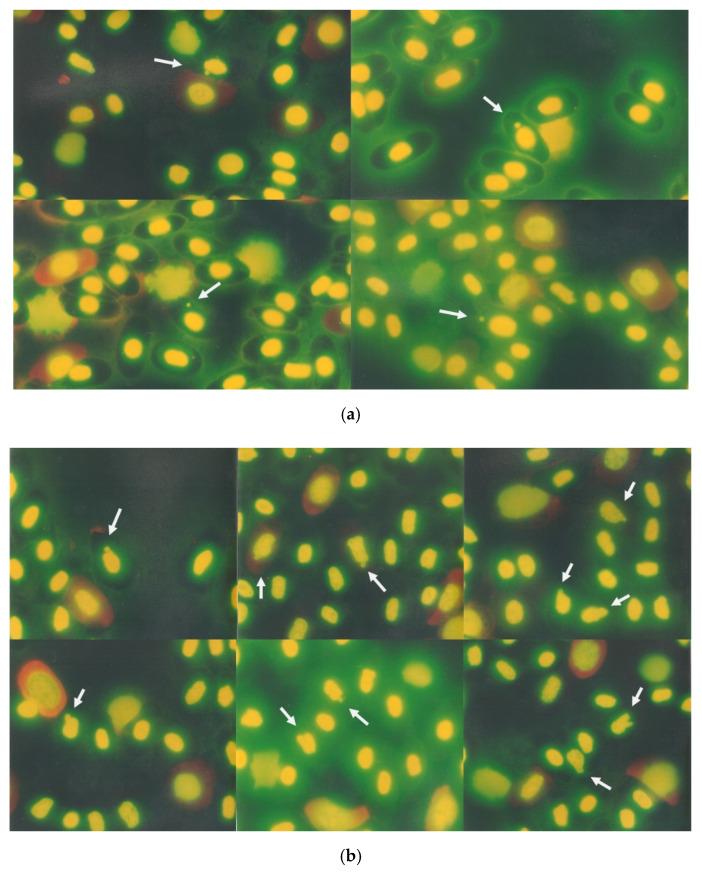
Erythrocytes with micronuclei ((**a**) above) and nuclear buds ((**b**) down) in peripheral crocodile blood sample were observed.

MNEs were counted only when the micronucleus was clearly separated from the main nucleus and had a round shape (Figure 1a). NBEs were counted when an elongation originated from the nucleus, partially overlapping the nucleus, or it was in the form of a drop with an obvious (or presumed) strand connecting it to the nucleus (Figure 1b).

### 2.5. Ethical Considerations

This research project was approved by the Ethics and Research Committee of the Centro de Investigación Biomédica de Occidente, Instituto Mexicano del Seguro Social, Guadalajara, Jalisco, Mexico, in accordance with the Provisions for the Use and Care of Experimental Animals [29,30] and International Health Institutes for the Humane Treatment of Research Animals to comply with the Animal Research: Reporting of In Vivo Experiments (ARRIVE) guidelines [31,32].

The organisms used in this study had the permits and legal requirements established in the General Law of Ecological Balance and Environmental Protection, as well as in the General Law of Wildlife and the Convention on International Trade in Endangered Species of Wild Fauna and Flora (CITES). The destination of the specimens was determined by the Ministry of Environment and Natural Resources—SEMARNAT, a federal government agency in charge of issuing the corresponding licenses and authorizations.

### 2.6. Statistical Analysis

The data were evaluated using the Statistical Package for Social Science software v.18.0 (SPSS, IBM Co., Armonk, NY, USA), and all data were expressed as the mean ± standard deviation. The results were assessed by means of an analysis of variance (ANOVA) for repeated measures and the LSD adjustment test for multiple post hoc comparisons, which were considered statistically different when the *p*-value was less than 0.05.

## 3. Results

**Figure 2:**
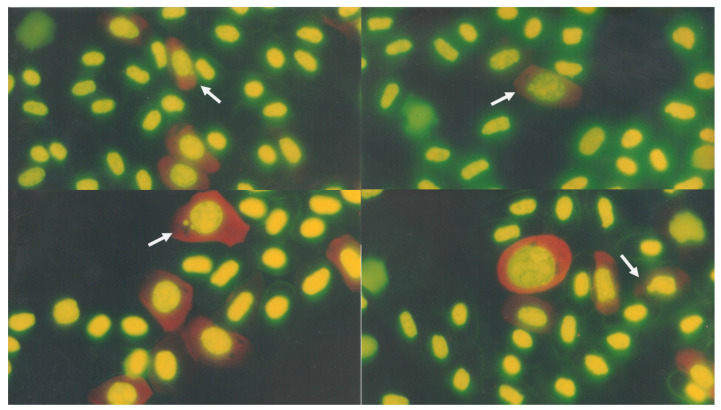
Young erythrocytes with micronuclei and nuclear buds in peripheral crocodile blood samples were observed.

Table 1 shows the values of the organisms, which include counts of MNEs, NBEs, MNYEs, NBYEs, and the YE proportion; these structures are shown in Figure 1a,b and Figure 2.

No significant differences were observed in the YE proportion values in sampling days. Similarly, the controls did not show significant differences for MNEs, MNYEs, as well as for NBEs and NBYEs measurements. The 7 mg group showed significant differences only in MNEs at 48 h (*p <* 0.04) and 72 h (*p* < 0.03).

**Figure 3:**
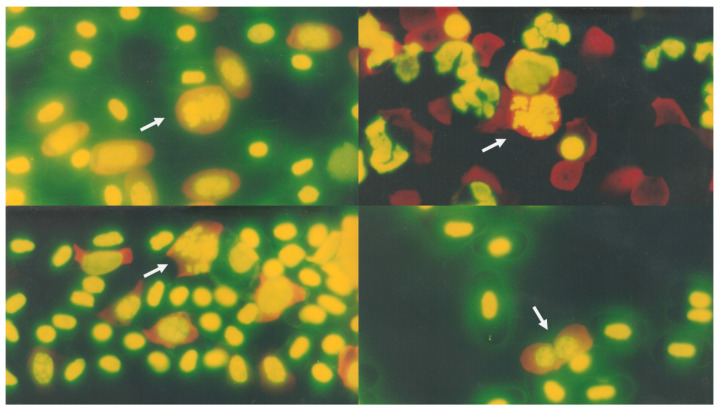
Crocodile blood smears: erythrocytes were observed in mitosis.

Regarding nuclear extensions, significant differences were observed in the 7 mg/Kg group in NBEs at 120 h (*p* < 0.03), while in the 10 mg group, significant differences were observed in NBEs at 24 h (*p* < 0.01), 48 h (*p* < 0.001), 72 h (*p* < 0.01), and 96 h (*p* < 0.03), and in NBYEs at 72 h (*p* < 0.05) and 96 h (*p* < 0.01). Additionally, some mitotic activity in circulation was observed in immature erythrocytes (Figure 3).

## 4. Discussion

Crocodiles have been used as biomonitoring models to detect the presence of total dichlorodiphenyltrichloroethane (DDT) in peripheral blood since they can adapt to underwater and dry land-living conditions [33]. In the same way, caimans have been shown to be good models in toxicity studies by exposing newborn specimens in the water [34] or treating the eggs before hatching by inducing damage to the genetic material [27,35].

In the present study, the frequencies of MNEs, NBEs, MNYEs, NBYEs, and the proportion of YEs were measured in *C. moreletii* to determine if these parameters could be informative according to the genotoxic effects observed in vivo as they were previously observed in pig and human lymphocyte cultures, parrot peripheral blood erythrocytes [17,20] or in reptiles like caimans [27,28,34,35,36].

Considering the advantage of using bioindicators to assess DNA damage, particularly by means of the micronucleus assay, crocodiles produce spontaneous MN, so when exposed to genotoxic agents, an increase in MN should be observed due to their accumulation since the spleen lacks the ability to quickly eliminate them from circulation.

In this study, crocodiles present spontaneous MNEs and NBEs. The NBE frequencies were higher than MNEs (Table 1), suggesting that NBEs may be more sensitive for detecting a greater spectrum of DNA damage than MNEs, or both structures could be used to complement the monitoring process. This could be explained by the formation mechanism of NBs and MN since it has been described that NBs probably give rise to MN [18,37]. This finding could also be attributed to the fact that MN can be readily eliminated from the circulation by the spleen as they are not connected to the main nucleus. This cleaning system recognizes an erythrocyte with MN as an abnormal cell, so once it passes through the spleen, it is identified as abnormal and destroyed. Possibly this does not occur with NBs, since they keep a union with the main nucleus, so they are not recognized as foreign bodies embedded in the cytoplasm as in the case of MN.

Factors such as general health and nutritional status, sex, age, and environmental contamination are important variables that influence chromosomal damage and cell proliferation [38]. In the present work, all organisms presented an apparent good state of health at the time of the experiment. Even though all crocodiles were maintained under the same conditions before the study to eliminate the possible influence of prior genotoxic exposure, the crocodiles used in the present study were also kept under quarantine for a minimum of 9 days before the experiments.

CP was administered to swamp crocodiles once a day for two days at low doses (a total of 14 and 20 mg/kg of weight, respectively). When in rodents, the recommended dose for the positive control is 50 to 60 mg/kg of weight. This was performed only with the intention of observing the behavior of both MNEs and NBEs parameters through a very slight micronucleogenic stimulus and thus determine if the same effect could be detected by means of monitoring NBEs as they could be more sensitive than MN.

The importance of this assay is that in previous studies, a greater sensitivity has been observed in detecting nuclear abnormalities different from MN, meaning that with less damage, an increase in nuclear abnormalities may be observed before an MN increase [21,22].

Only cells with MN or NBs were analyzed herein, and the presence of other nuclear abnormalities, such as binucleated cells, karyolysis, pyknosis, karyorrhexis, and condensed chromatin as nuclear fragmentation, was not considered. However, both MN and NBs are related to genetic material damage of organisms, and at least under these conditions, the presence of NBs showed a greater significant difference in the different times after the administration of CP than MN frequency, which is in accordance with other studies [21,22].

Unlike previous studies, the present work ensured counts were performed with a greater number of cells (1000 with YEs and 10,000 in TEs vs 500 cells counted in other studies); also, the proportion of YEs was also considered to have control of the system (if the proportion of YEs decreases, it means that the bone marrow that is in charge of its production is suffering from myelosuppression, and as a result, the organism is no longer informative). Additionally, the analysis of YE frequency was included because this parameter indicates recent damage. In this experiment, to ensure the adequate administered dose, a cannula of 8 cm in length was used to deposit the compound directly in the stomach (in the studies reported with caimans, the dosage was carried out directly in the eggs before hatching or in the water where newborn crocodiles stayed). The sample was obtained by cutting the claw near the base of the phalanx to obtain the blood drop required for the sample (in other studies, the sample was obtained from the vein), and lastly, the staining was carried out with acridine orange, which allowed YEs to be differentially stained from the mature erythrocytes.

Crocodiles are reptiles whose erythrocytes are large and nucleated and have abundant cytoplasm, and these characteristics facilitated the observation of MNEs and NBEs. These organisms also spend time on Earth and in the water, which made them suitable bioindicators that could be used to determine environmental genotoxic damage in both environments with just a drop of blood.

Taken together, these results indicate that NBEs could be an alternative parameter in genotoxicity studies in model species with nucleated erythrocytes or when it is difficult to establish a spontaneous MNE frequency.

A variable to consider in the present work was the temperature since crocodiles are poikilothermic organisms, also called exothermic or “cold-blooded”; that is, they cannot significantly regulate their body temperature by generating heat, and this varies according to the temperature of their environment [33]. In the present work, a system was developed to maintain a constant temperature of 30 °C inside the crocodile shelter where animals were kept during the experimental periods. Temperature is an important environmental factor since it exerts several effects, for instance, on the feeding behavior and digestive process of reptiles and on the DNA of cells in an organism [39]. In relation to the effect on feeding behavior, most crocodiles stop feeding when the temperature drops below 25 °C, which is why the optimum temperature for feeding is between 25 and 35 °C.

Regarding the effect on genetic material, we know that exposure to high temperatures increases DNA damage since this macromolecule is denatured at 70 °C. For example, in mice exposed to temperatures above 39.5 °C, an increase in MN was observed in the bone marrow [40]. In this context, the literature describes that temperature exerts numerous effects on cells of living organisms [39], including MN [41]. One of the mechanisms of action proposed in which the increase in temperature affects DNA is that the temperature change disrupts the fibers of the mitotic spindle [40], which is classified as an aneuploidogenic agent. Therefore, in the present work, the temperature at which crocodiles were kept in their shelter during the time of the experiment was 30 °C to ensure that this variable would not affect the results and coupled with the fact that it is the average temperature of the collection site of organisms. With respect to the observation of immature erythrocytes in crocodile blood smears, one explanation is that reptile red blood cells are produced in the bone marrow, or under certain conditions, in the spleen and liver, often culminating in their maturation in circulating blood. This makes it possible to find circulating immature erythrocytes, such as rubricytes, YEs, or reticulocytes, in mitosis, especially in young crocodiles [42,43]. A phenomenon that has been observed only in samples of premature children in previous studies, which are considered immature organisms.

**Figure 4:**
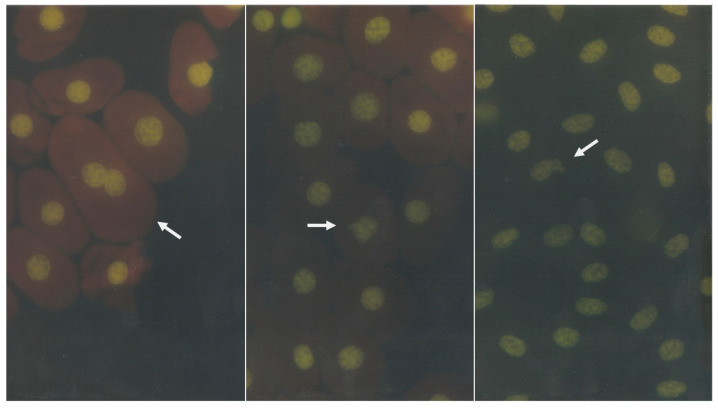
Erythrocytes with nuclear abnormalities in peripheral blood samples from reptiles were observed. Left shows a binucleated cell from a turtle; center shows a nuclear bud from a turtle; and right shows a nuclear bud from a rattlesnake.

It is important to consider that, of the five classes of chordates (reptiles, mammals, amphibians, fish, and birds), only mammals do not present erythrocytes with nuclei; thus, the species with nucleated erythrocytes represent most species with research possibilities of this kind. For example, nuclear abnormalities can be observed (Figure 4) in erythrocytes of the rattleshield Rattlesnake (*Crotalus molossus*) and lizard turtle (Alligator snapping turtle, *Macroclemys temminckii*).

In crocodiles, the formation of NBEs, as well as MNEs, could be induced due to the effect observed in nucleated peripheral blood erythrocytes when exposed to two different doses of CP every 24 h for two days. In the case of MNEs, increased numbers of these structures were observed in the study groups exposed to one of the two doses of CP compared to the baseline value, and these increases were significant in the group that received CP at a dose of 7 mg/kg.

Similarly, the number of NBEs increased in the groups exposed to CP, and this change was significant in the group that received the 10 mg/Kg dose. The fact that the increase in the number of MNEs reached a statistical difference in the 7 mg/Kg dose of the CP group, and the number of NBEs in the group that received the 10 mg/Kg dose was most likely due to the high standard deviations found in the results; thus, the increased numbers observed in the exposed groups did not reach significance, possibly because the quarantine period of 9 days was insufficient considering these organisms were not obtained from a vivarium, and parameters, such as sex and nutritional status, are variables that may influence the number of MNs [44]. In the group of 10 mg/Kg of CP, a slight decrease in YEs is appreciated at 48 h, although it was not significant; however, it allows establishing that even at this dose, a slight effect on cellular division in the crocodile is observed at 48 h. In this experiment, the study groups were formed independently of sex, with organisms of a similar weight and size, as well as the same time and place of collection.

In the MN assay using peripheral blood erythrocytes, the proportion of circulating YEs determines if the dose used is cytotoxic and produces myelosuppression (this also defines whether there is still cell division), which could lead to a wrong conclusion and a false negative result due to the action of genotoxic agents. Furthermore, the concentrations used of the genotoxic agent or the susceptibility of the species can even cause YEs to decrease to levels close to zero and the consequent drop in the number of MNEs due to the cytotoxicity caused by genotoxic agents [9,23]. Herein, we used two low doses of CP to compare our results to those reported as positive controls in other studies to demonstrate if the number of NBs would increase more than MN frequency.

In this study, we ruled out the cytotoxic effect of CP since the proportion of YEs was not affected in the CP study groups. As previously mentioned, the swamp crocodiles were collected in the same place and time of sampling; once they arrived at the laboratory, they were quarantined to adapt to controlled laboratory conditions. One of the important parameters of the adaptation process to the environment for organisms is feeding, a procedure that was carried out using rat and mouse neonates provided by the Institutional animal facility. Therefore, once crocodiles passed the adaptation period and were eating properly, they were included in the study. Newborn rats previously sacrificed by decapitation were used as food since it was not convenient to use any chemical product as the intention was to avoid adding more variables to the experiment that could affect the results.

In the present work, we observed that, as in other species, monitoring YE values in crocodiles is useful to evaluate the physiological state and promptly detect if the organism is in optimal conditions to monitor its state during the sampling time and to analyze the conditions of their habitats since the MN test requires tissues in constant division. Additionally, when analyzing the crocodile blood smears, erythrocytes were observed in mitosis (Figure 3), an uncommon event in chordates, since cell division is shown in blood circulation.

The evaluation of MN and NBs as a genotoxic assay is a quick, simple, and inexpensive test, with good results since these parameters indicate that the greater the number of MN or NBs, the greater damage to the genetic material. Another advantage of these in vivo tests in peripheral blood erythrocytes is that only a sample of blood is required to perform the smears. In the present work, the sample was obtained by cutting the claw, which is a simple, fast, and less invasive procedure that facilitates the testing process.

In the present work, CP was able to induce an increased number of NBEs, MNEs, NBYEs and MNYEs, which were used to evaluate the long- and short-term damage in nucleated erythrocytes of the swamp crocodile. Similar increases in the number of NBs have been previously described in cell cultures [17].

## 5. Conclusions

NBEs can be a useful complementary tool to monitor genotoxicity when working with species that have nucleated erythrocytes or when it is not possible to establish MNE frequency due to its advantages as a fast, simple, and inexpensive test with reliable results that only requires a drop of blood since NBs and MN are translated in the cellular sphere as damage to the genetic material, and they may be beneficial in studies where the MN test is also feasible, so these parameters could be useful to analyze the in vivo health status of this reptiles and for biomonitoring genotoxic pollutants in their habitats.

## Figures and Tables

**Table 1:** Effect of cyclophosphamide on the number of young erythrocytes (YEs), micronucleated erythrocytes (MNEs), erythrocytes with nuclear buds (NBEs), young erythrocytes with nuclear buds (NBYEs) and micronucleated young erythrocytes (MNYEs) in the swamp crocodile.

			Sampling Times					
Group	*n*	Parameter Analyzed	0 h	24 h	48 h	72 h	96 h	120 h
Group 1 Negative control (500 µL injectable water)	5	YEs/1000 TEs	24.2 ± 14.1	31.8 ± 18.3	29.2 ± 17.0	37.2 ± 14.2	23.0 ± 6.67	18.0 ± 7.34
				NS	NS	NS	NS	NS
		MNEs/10,000 TEs	3.0 ± 1.8	4.0 ± 3.0	4.0 ± 2.8	4.0 ± 3.39	4.8 ± 5.93	2.6 ± 2.0
				NS	NS	NS	NS	NS
		MNYEs/1000 YEs	1.0 ± 1.4	2.0 ± 1.8	0.6 ± 0.5	0.4 ± 0.8	0.2 ± 0.4	0.4 ± 0.5
				NS	NS	NS	NS	NS
		NBEs/10,000 TEs	52.2 ± 21.8	41.4 ± 9.7	39.0 ± 7.5	50.6 ± 20.3	53.0 ± 16.4	56.0 ± 22.7
				NS	NS	NS	NS	NS
		NBYEs/1000 YEs	13.2 ± 5.2	15.4 ± 6.8	21.2 ± 13.8	18.8 ± 10.0	15.0 ± 11.0	14.4 ± 13.0
				NS	NS	NS	NS	NS
Group 2 (7 mg/Kg de CP)	5	YEs/1000 TEs	15.2 ± 10.9	17.0 ± 11.8	16.8 ± 14.0	17.4 ± 8.5	14.2 ± 11.23	11.60 ± 6.42
				NS	NS	NS	NS	NS
		MNEs/10,000 TEs	3.4 ± 2.6	5.8 ± 2.8	10.4 ± 7.5	12.0 ± 8.3	7.0 ± 5.1	4.6 ± 2.9
				NS	*p* < 0.04	*p* < 0.03	NS	NS
		MNYEs/1000 YEs	0.6 ± 0.8	0.8 ± 0.8	1.0 ± 1.4	0.4 ± 0.5	0.6 ± 0.8	0.4 ± 0.5
				NS	NS	NS	NS	NS
		NBEs/10,000 TEs	46.0 ± 14.4	43.6 ± 26.9	45.0 ± 16.5	75.6 ± 41.2	85.8 ± 12.1	105.6 ± 19.9
				NS	NS	NS	NS	*p* < 0.03
		NBYEs/1000 YEs	27.8 ± 20.5	18.6 ± 8.9	18.0 ± 6.8	14.2 ± 6.3	12.6 ± 6.5	18.4 ± 16.9
				NS	NS	NS	NS	NS
Group 3 (10 mg/Kg de CP)	5	YEs/1000 TEs	20.8 ± 8.34	18.6 ± 9.0	12.6 ± 5.3	19.4 ± 8.0	20.0 ± 7.5	23.8 ± 7.4
				NS	NS	NS	NS	NS
		MNEs/10,000 TEs	3.8 ± 2.5	4.4 ± 4.2	5.8 ± 4.2	5.8 ± 3.2	3.4 ± 2.7	5.8 ± 3.3
				NS	NS	NS	NS	NS
		MNYEs/1000 YEs	1.2 ± 1.3	0.4 ± 0.8	0.8 ± 1.3	1.2 ± 1.3	1.8 ± 1.4	0.6 ± 0.5
				NS	NS	NS	NS	NS
		NBEs/10,000 TEs	70.0 ± 11.1	107.2 ± 10.4	108.8 ± 8.5	105.2 ± 10.5	96.8 ± 10.4	98.0 ± 27.5
				*p* < 0.01	*p* < 0.001	*p* < 0.01	*p* < 0.03	NS
		NBYEs/1000 YEs	17.0 ± 6.7	30.8 ± 13.6	35.6 ± 15.6	36.2 ± 11.8	32.2 ± 5.35	21.0 ± 12.1
				NS	NS	*p* < 0.05	*p* < 0.01	NS

The results are expressed as mean ± standard deviation. MNEs: micronucleated erythrocytes; MNYEs: micronucleated young erythrocytes; NBEs: erythrocytes with nuclear buds; NBYEs: young erythrocytes with nuclear buds; YEs: young erythrocytes; TEs: total erythrocytes; CP: cyclophosphamide; NS: not significant; *n*: sample size. Comparisons were performed in each group between the baseline value (0 h) with the following sampling times (24, 48, 72, 96 and 120 h) for each analyzed parameter. Statistical significance was set at a *p*-value less than 0.05. All doses were adjusted to a final volume of 500 µL with injectable water.

## Data Availability

The data that support the findings of this study are available from the corresponding author (B.C.G.-M.) upon reasonable request.
